# Novel experimental *Pseudomonas aeruginosa* lung infection model mimicking long-term host–pathogen interactions in cystic fibrosis

**DOI:** 10.1111/j.1600-0463.2008.00018.x

**Published:** 2009-02

**Authors:** CLAUS MOSER, MARIA VAN GENNIP, THOMAS BJARNSHOLT, PETER ØSTRUP JENSEN, BAOLERI LEE, HANS PETTER HOUGEN, HENRIK CALUM, OANA CIOFU, MICHAEL GIVSKOV, SØREN MOLIN, NIELS HØIBY

**Affiliations:** 1 Department of Clinical Microbiology, Copenhagen University HospitalRigshospitalet, Copenhagen; 2 Technical University of DenmarkCopenhagen; 3 Institute of International Health, Immunology and MicrobiologyCopenhagen, Denmark; 4 Department of Forensic Medicine, University of CopenhagenCopenhagen, Denmark

**Keywords:** *Pseudomonas aeruginosa*, cystic fibrosis, adaptation, chronic lung infection, host response

## Abstract

The dominant cause of premature death in patients suffering from cystic fibrosis (CF) is chronic lung infection with *Pseudomonas aeruginosa*. The chronic lung infection often lasts for decades with just one clone. However, as a result of inflammation, antibiotic treatment and different niches in the lungs, the clone undergoes significant genetic changes, resulting in diversifying geno- and phenotypes. Such an adaptation may generate different host responses. To experimentally reflect the year-long chronic lung infection in CF, groups of BALB/c mice were infected with clonal isolates from different periods (1980, 1988, 1997, 1999 and 2003) of the chronic lung infection of one CF patient using the seaweed alginate embedment model. The results showed that the non-mucoid clones reduced their virulence over time, resulting in faster clearing of the bacteria from the lungs, improved pathology and reduced pulmonary production of macrophage inflammatory protein-2 (MIP-2) and granulocyte colony-stimulating factor (G-CSF). In contrast, the mucoid clones were more virulent and virulence increased with time, resulting in impaired pulmonary clearing of the latest clone, severe inflammation and increased pulmonary MIP-2 and G-CSF production. In conclusion, adaptation of *P. aeruginosa* in CF is reflected by changed ability to establish lung infection and results in distinct host responses to mucoid and non-mucoid phenotypes.

The majority of adult patients with the inherited disease cystic fibrosis (CF) have acquired chronic *Pseudomonas aeruginosa* lung infection, due to decreased airway fluid, resulting in reduced ciliary clearance of aspirated microbes (1). The induced host response is characterized by an influx of numerous polymorphonuclear neutrophil granulocytes (PMNs) and an induction of a Th2-dominated response with pronounced antibody and IL-4 production (2–4). However, the chronic *P. aeruginosa* lung infection resists the host response, as well as antibiotic treatment to eradicate the microorganisms from the lungs (5, 6). The persistence of the infection is ascribed to the ability of *P. aeruginosa* to form biofilms, where bacteria grow in microcolonies in a self-produced extracellular polymeric matrix, to mutate to mucoid phenotypes hyperproducing an exopolysaccharide called alginate and to develop resistance to antibiotics by becoming mutators (6–8). The inflammation induced by the chronic *P. aeruginosa* lung infection leads to a gradual degradation of the lung tissue due to PMN proteases and reactive oxygen species from the PMNs (6, 9, 10).

Although the dominant outcome of the lung infection in CF is tissue damage and premature death or lung transplantation, there is a significant effect of the inflammation on *P. aeruginosa* residing in the lungs (6). The result is a year-long interplay between the host and the pathogen unless the infecting strain is replaced by a more fit strain, which occasionally is the case (11). Although replacement of a dominant strain can take place, it is believed that CF patients are infected with one clone for several years – often decades (6, 11). However, even though the patients are infected with one clone, several different phenotypes are present due to adaptation (6). The general background and consequences of adaptation and diversity have received considerable attention from environmental microbiologists (12, 13). Early in the course of disease, intermittent colonization with one fit, often environmental and physiologically adaptable clone takes place. In later stages of infection, genomic adaptation can dominate, e.g. in the case of hypermutators (14–17).

In the case of *P. aeruginosa* and CF, adaptation has been demonstrated to be involved already from the initial phases during the interplay between pathogen and epithelia (18), and continue during infection (19, 20). This is reflected in approximately 10% larger genome of clinical *P. aeruginosa* strains as compared with environmental strains and the PAO1 type strain (21). Another example of the adaptation of *P. aeruginosa* during the 20–30 years of chronic lung infection in CF was demonstrated by Lee et al. (22), who investigated the ability of *in vitro* biofilm formation of pulsed-field gel electrophoresis (PFGE) identical non-mucoid clinical strains from CF patients. Biofilm formation significantly changed over time, and the finding was that the ability of biofilm formation decreased from the early isolates to the late isolates (22). In addition, changes in the quorum-sensing (QS) status and the production of exoproteases were observed, and the appearance of hypermutable strains seemed to increase with the duration of the lung infection (22). The diversity with different phenotypes and genotypes following the initial phases probably reflects the adaptation to different niches of the lungs and the subsequent higher orders of complexity result in increased fitness of the strain that infected the CF patient (6, 16, 17). Indeed, the induced inflammation and subsequent tissue destruction may generate more spatially heterogeneous niches with different physiologies (e.g. changed levels of oxygen) for adapting mutators or recombinants (17, 23).

Animal models mimicking the adaptation during chronic *P. aeruginosa* lung infection are pivotal to further improve our understanding of the patho-physiological mechanisms during the persistent infection in CF and related diseases like diffuse panbronchiolitis. However, no known animal model reflects the 20–30 years of bacterial–host interplay observed in CF. No model reflects the fact that clones of *P. aeruginosa* isolated at the early stages of the chronic infection behave significantly different from the same clones isolated at later or terminal stages of the chronic lung infection thousands of bacterial generations later. Such evolution of the bacterial clones may induce completely different host responses, which again may indicate the need for different treatment strategies.

Based on those observations, we aimed at establishing a new experimental strategy infecting different groups of BALB/c mice with PFGE-identical mucoid and non-mucoid isolates from the same CF patient isolated during different periods of her chronic lung infection for 23 years. The experiments were evaluated by quantitative bacteriology, macroscopic and microscopic pathology, as well as pulmonary cytokine production.

## MATERIAL AND METHODS

### Mice

Female BALB/c mice, 11 weeks of age, were purchased from M&B Laboratory Animals (Ry, Denmark), and given unlimited access to chow and water. Mice were left to acclimatize for 1 week before the experiments were performed, and all the experiments were performed under the guidelines of the National Ministry of Justice.

### CF patient

**Fig. 1:**
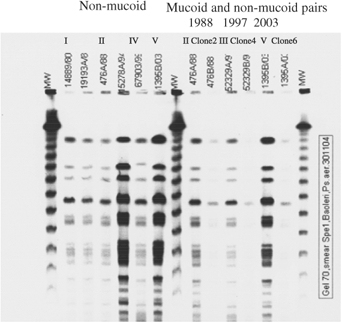
Pulsed-field gel electrophoresis (PFGE) typing of the *Pseudomonas aeruginosa* clones. PFGE typing of the *P. aeruginosa* clones from one cystic fibrosis patient chronically infected for 23 years. All non-mucoid clones (I, II, IV and V) [to the left from the middle molecular weight latter (MW), III to the right] and the mucoid (marked clone 2, 4 or 6), non-mucoid pairs (to the right from the middle MW latter) had the same pulsed-field gel electrophoresis patterns.

The set of clonal *P. aeruginosa* strains, on which the present animal model is based, was from a female CF patient born in 1966. She has a heterozygote mutation ΔF508/3128 del4. She has been chronically infected with *P. aeruginosa* since 1971, and had a high number of precipitating anti-pseudomonas antibodies. However, the isotypic clones included in the present study were cultured in 1980 for the first time. In 2000, the patient underwent a double-lung transplant, but was again chronically infected within a few months with the same isotypic clone. The PFGE patterns are shown in Fig. 1.

### Bacterial strains

All CF patients at the Copenhagen CF centre are seen on a monthly basis, and provide a sputum sample for microscopy and culture. Strains from all patients are frozen at −80 °C on a regular basis. The non-mucoid clonal isolates used in the present study were used in a recently published study (22), and were isolated in 1980 and 1988 (early isolates), and 1997, 1999 and 2003 (late isolates). The mucoid clonal isolates are from the same sputum samples as the non-mucoid clonal isolates from 1988, 1997 and 2003. To maintain consistency in our findings with the clone collection, three non-mucoid PFGE identical isolates from two CF patients, as well as two pairs of early and late isotyped mucoid isolates from two other chronically infected CF patients were included in the study. Infection of mice with these control isolates from different time periods were evaluated by quantitative bacteriology and macroscopic pathology, and mortality was registered.

PFGE analysis was performed as described previously (24). Evaluation of similarity was performed as described by Tenover et al. (25).

**Fig. 3:**
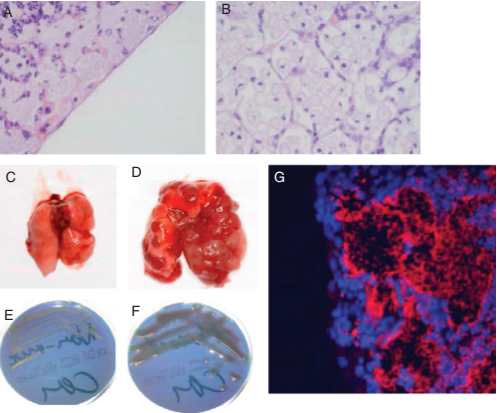
Pathology and clonal phenotypes. Microscopic pictures haematoxylin and eosin (HE staining, Panel A × 600 and B × 1000) of lungs of mice infected with the late mucoid *Pseudomonas aeruginosa* from 2003. Macroscopically affected lung parts were isolated and fixed in formaldehyde. Embedded in wax and 5-μm-thick sections cut for HE staining. In panel A, alveoli next to the pleural cavity can be seen completely filled up with bacteria. Polymorphonuclear neutrophil granulocytes (PMNs) can be seen penetrating into the airway lumen. In Panel B (HE, × 1000), alveoli are filled up with swollen cells, with small nuclei, which may represent macrophages having phagocytized dead PMNs and therefore contain high amounts of lipids. Macroscopic pictures of lungs infected with mucoid *P. aeruginosa* 0.8 × 10^6^ colony-forming units per mouse (early isolate from 1988 panel C and late isolate from 2003 panel D) using the seaweed alginate embedment model. Mice were sacrificed 5 days after infection. In panel D, the lungs are dominated by macro-abscesses and haemorrhagic areas, with minor areas of normal lung tissue in between. To the left is a lung, that has cleared an infection with a low dose of an early mucoid *P. aeruginosa* isolate. No affected areas are seen. For comparison of alginate production, panels E and F show photos of a non-mucoid and a mucoid *P. aeruginosa* pair included in the study. In panel G, fluorescence microscopy pictures (× 600) of peripheral mouse airways stained with *P. aeruginosa*-specific Peptide nucleic acid (PNA)-fluorescence *in situ* hybridization (FISH) (red) and 4′,6-diamidino-2-phenylindole (blue) can be seen. The lungs have been infected with the late mucoid 2003 isolate. Only areas with bacteria-like structures stained red, and alveoli are seen filled up with *P. aeruginosa*.

Colonies of the late non-mucoid (Fig. 3E) and mucoid isolates from 2003 are shown for comparison.

### QS

Production of acyl-homoserine lactones (AHL) was detected in the supernatant from overnight bacterial cultures using the AHL-specific reporter strains as described by Hentzer et al. (26).

### Challenge procedure

Immobilization of bacteria was performed as described previously (27). Briefly, one colony was added to 100 ml sterile filtered oxbroth, and cultured at 37 °C for 18 h on a gyratory shaker. The overnight culture was centrifuged at 4 °C and 4400 *g*. The supernatant was discarded and the pellet was resuspended in 5 ml sterile serum bouillon [Statens Seruminstitut (SSI), Copenhagen, Denmark]. One millilitre of the bacterial suspension was mixed with 9 ml sterile seaweed alginate suspension (11 mg/ml of 60% guluronic acid protanal 10/60 (Protan, Drammen, Norway) dissolved in 0.9% NaCl). The suspension was placed in a cylindrical reservoir and forced through an 18 G cannula, with a coaxial jet of air blowing on the alginate droplets. The alginate droplets were collected in a solution of 0.1 M CaCl_2_ Tris-HCl buffer (0.1 M, pH 7.0). After 1 h of stirring, the alginate beads were washed twice in 0.9% NaCl. The colony-forming units (CFU) were controlled by serial dilution and cultured on a modified Conradi-Drigalski medium (SSI) selective for Gram-negative bacteria. Based on dose–response experiments, the suspension was adjusted to 10^8^ CFU/ml for the non-mucoid isolates and confirmed by colony counts (corresponding to a challenge dose of 4 × 10^6^ CFU per mouse). At this challenge dose, the early isolates from 1980 or 1988 revealed a significantly higher, but acceptable mortality rate at 20–25% as compared with the late isolates (p<0.025). In the study comparing the outcome after infection with mucoid or non-mucoid isolates, no significant differences were observed between the early mucoid isolate and the three non-mucoid isolates (data not shown). However, survival was significantly reduced when mice were infected with either of the late mucoid isolates from 1997 or 2003, because only two mice survived in the 2003 group (p<0.005). Therefore, the challenge dose for the mucoid isolates was reduced to 0.8 × 10^6^ CFU per mouse.

At the time of the challenge, mice were anaesthetized subcutaneously with a 1:1 mixture of etomidate (Jannsen, Birkeroed, Denmark) and midazolam (Roche, Basel, Switzerland) (10 ml/kg body weight), and tracheotomized (28). An intratracheal challenge with 0.04 ml of *P. aeruginosa* embedded in seaweed alginate beads was performed with a bead-tipped needle. The inoculum was installed in the left lung 11 mm from the penetration site (28).

Pentobarbital (DAK, Copenhagen, Denmark) 2.0 ml/kg body weight was used to sacrifice the animals (28). Mice were sacrificed at day 5, because both the innate and the adaptive immune responses are activated at this time point (own observation).

### Macroscopic pathology

Upon sacrifice, macroscopic signs of pathology were noted. A broad estimate of the expansion of the affected areas was registered as a fraction of the referred lung lobe. All estimates were performed blinded.

### Histopathology

The lungs were prepared for histopathological examination as described previously (28). Briefly, the affected lung was fixed in a 4% w/v formaldehyde solution (Bie & Berntsen, Copenhagen, Denmark) embedded in paraffin wax and cut into 5-μm-thick sections, followed by haematoxylin and eosin staining. The entire lung slide was scanned at a low magnitude, and from an average evaluation of a minimum of five representative areas at higher magnitude (× 500) the type of lung inflammation was estimated. The inflammatory responses were scored as acute (>90% PMNs), chronic [>90% mononuclear cells (MN)], both types present, neither dominating (PMN/MN) or no inflammation (NI) (28). The degree of inflammation was scored on a scale from 0 to 3+, where 0 means NI, + means mild focal inflammation, ++ mean moderate to severe focal inflammation and +++ means severe inflammation to necrosis, or severe inflammation throughout the lung. In addition, the presence of atelectasis or micro-abscesses was noted. The histopathological evaluation was performed blinded.

### Peptide nucleic acid (PNA)-fluorescence *in situ* hybridization (FISH)

To confirm the nature of bacteria-like structures in the alveoles, deparaffinized tissue sections were analysed by FISH using PNA probes. A mixture of a Texas Red-labelled, *P. aeruginosa*-specific PNA probe and a fluorescein isothiocyanate (FITC)-labelled, universal bacterium PNA probe in a hybridization solution (AdvanDx Inc., Woburn, MA, USA) was added to each section and hybridized in a PNA-FISH Workstation at 55 °C for 90 min covered by a lid. The slides were washed for 30 min at 55 °C in Wash Solution (AdvanDx Inc.). Vectashield mounting media with 4′,6-diamidino-2-phenylindole (DAPI) (Vector laboratories, Burlingame, CA, USA) was applied, and a cover slip was added to each slide. Slides were read using a fluorescence microscope equipped with an FITC, a Texas Red and a DAPI filter.

### Quantitative bacteriology

Lungs for quantitative bacteriology were prepared as described previously (28). In brief, the lungs were removed aseptically and homogenized in 5 ml of PBS and serial dilutions of the homogenate were plated, incubated for 24 h and the numbers of CFU were determined and presented as log CFU per lung.

### Cytokine production

The lung homogenates were centrifuged at 4400 *g* for 10 min and the supernatants were isolated and kept at –70 °C until cytokine analysis. The concentrations in the lung homogenates of the PMN mobilizer granulocyte colony-stimulating factor (G-CSF) and the PMN chemoattractant and murine IL-8 analogue macrophage inflammatory protein-2 (MIP-2) were measured by ELISA (R&D, Minneapolis, MN, USA) according to the manufacturer's instructions.

### Statistical analysis

The number of mice in each group was calculated to provide a power of 0.80 or higher for continuous data. Statistical calculations were performed using Statview (Abacus Concepts, Berkeley, CA, USA). The χ^2^ test was used when comparing qualitative variables, and the ANOVA/unpaired t-test was used when comparing quantitative variables. p≤0.05 was considered statistically significant.

## RESULTS

### QS

As can be seen from Table 1, the early non-mucoid strain from 1988 produced both AHL, C4 and C12. The same was observed from the early and the intermediate mucoid strains. The late mucoid strain from 2003 produced only the C12 AHL.

**Table 1:** Production of acyl-homoserine lactones in three PFGE-identical pairs of mucoid and non-mucoid *Pseudomonas aeruginosa*

Clonal isolates	C4	C12
Non-mucoid 1988	+	+++
Mucoid 1988	+	+
Non-mucoid 1997	−	−
Mucoid 1997	+	+
Non-mucoid 2003	−	−
Mucoid 2003	−	+

Production of acyl-homoserine lactones (AHLs) by the mucoid clones (2, 4 and 6) and the non-mucoid clones (1, 3 and 5). Detected in supernatants from overnight bacterial cultures using AHL-specific reporter strains. Presented semiquantitatively from − (no detected production) to +++ (high production). Both the early mucoid and non-mucoid isolates from 1988 produced both quorum sensing signal molecules, whereas only the mucoid intermediate clone from 1997 produced both signal molecules. In contrast the late mucoid isolate from 2003 only produced the C12 signal molecule. Except for the early non-mucoid isolate, the non-mucoid clones did not produce quorum sensing signal molecules.

PFGE, pulsed-field gel electrophoresis.

The two late non-mucoid isolates were defective in producing both AHL.

### Quantitative bacteriology

#### Non-mucoid isolates

**Fig. 2:**
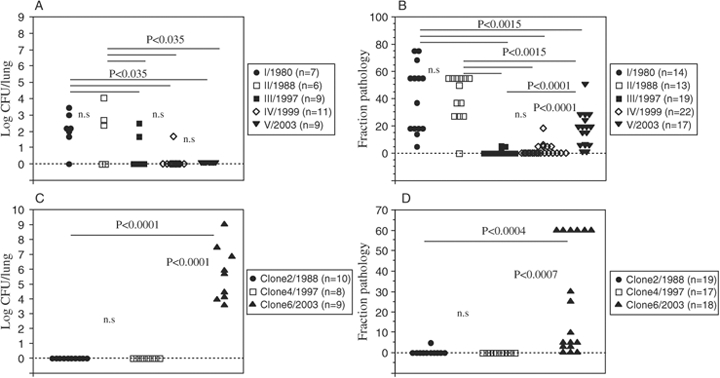
Quantitative bacteriology. Quantitative bacteriology of homogenized lungs from mice infected with non-mucoid [4 × 10^6^ colony-forming units (CFU) per mouse, panel A] or mucoid (0.8 × 10^6^ CFU per mouse, panel C) pulsed-field gel electrophoresis (PFGE)-identical *Pseudomonas aeruginosa* using the seaweed alginate embedment model. Evaluated day 5 after infection. A significantly faster clearance of the later non-mucoid clones was seen as compared with the early non-mucoid clones (p<0.035). In contrast, both the early and the intermediate mucoid isolates eradicated, whereas the late mucoid isolate was cultured from all mice, and even increased in number in four mice (p<0.0001). Macroscopic pathology. Macroscopic pathology was estimated as a relative affected area of the total lung area. Evaluated at day 5 after infection with non-mucoid [4 × 10^6^ colony-forming units (CFU) per mouse, panel B] or mucoid (0.8 × 10^6^ CFU per mouse, panel D) PFGE-identical *P. aeruginosa* using the seaweed alginate embedment model. The dissemination of macroscopic pathology decreased in mice infected with the later non-mucoid isolates (p<0.0015, panel B). In contrast, signs of macroscopic pathology were almost exclusively present in mice infected with the late mucoid isolate (p<0.0004, panel D). In addition, the observed changes in the lungs from mice infected with the late mucoid isolate were severe including macro-abscesses, adherences, haemorrhages and areas resembling air trapping.

Significantly higher CFUs were obtained from the groups of mice infected with any of the two early isolates as compared with the three groups infected with one of the late isolates (p<0.035; Fig. 2A).

#### Mucoid isolates

All mice infected with the late 2003 mucoid isolate had bacteria cultured from the lungs at day 5. In contrast, no bacteria were cultured from any mice infected with the early (1988) or the intermediate (1997) mucoid isolate. The difference was statistically significant (p<0.0001; Fig. 2C).

### Macroscopic pathology

#### Non-mucoid isolates

In infection with the non-mucoid isolates, only atelectasis was seen as a pathologic change. Dissemination of pathology was significantly increased in the two groups infected with one of the early isolates as compared with any of the three groups infected with one of the late isolates (p<0.0015; Fig. 2B). In addition, signs of pathology were significantly increased in the group infected with the latest (2003) isolate as compared with the groups infected with one of other two late (1997 and 1999) isolates (p<0.0001).

#### Mucoid isolates

Almost exclusively mice infected with the late mucoid isolate showed signs of macroscopic pathology. The dissemination of the affected areas was significantly increased as compared with the early (1988) isolate or the intermediate (1997) isolate (p<0.0004 and p<0.0007, respectively; Fig. 2D). In addition, the findings were severe with haemorrhages, abscesses, adherences and areas of air trapping in some of the mice (Fig. 3D). Mouse lung without any sign of inflammation can be seen in Fig. 3C for comparison.

### Histopathology

#### Non-mucoid isolates

In significantly more mice infected with one of the early 1980 or 1988 isolates, a PMN-involved inflammation was observed as compared with mice infected with the 1997 or the 1999 isolate (p<0.05; Table 2). This difference did not reach the level of significance as compared with the 2003 isolate (Table 2). The degree of inflammation was significantly worse in the mice infected with the early 1980 isolate as compared with mice infected with any of the late (1997, 1999 or 2003) isolates (p<0.02; Table 2). Mice infected with the 1988 isolate had a more severe degree of inflammation as compared with mice infected with the 1997 or the 1999 isolate (p<0.002; Table 2). The number of mice with atelectasis was increased in the groups infected with one of the early isolates from 1980 or 1988 (p<0.05; Table 2), although this did not reach the level of statistical significance when comparing 1988 with 2003 (p<0.1).

**Table 2:** Histopathology

Non-mucoid isolates				Mucoid isolates		
Groups	Type	Degree	Atelectasis	Type	Degree	Micro-abscess
I/1980	7 PMN/MN^*^	4++^**^	5 mice			
	0 MN	3+				
	0 NI	0−				
II/1988	7 PMN/MN^*^	2++^***^	4 mice	0 PMN	0+++	0 mice
	0 MN	5+		2 PMN/MN	0++	
	0 NI	0−		0 MN	2+	
				7 NI	7−	
III/1997	2 PMN/MN	0++	0 mice	0 PMN	0+++	0 mice
	0 MN	2+		0 PMN/MN	0++	
	8 NI	8−		0 MN	0+	
				9 NI	9−	
IV/1999	2 PMN/MN	0++	1 mouse			
	0 MN	2+				
	9 NI	9−				
V/2003	3 PMN/MN	1++	1 mouse	3 PMN^****^	3+++^****^	3 mice
	1 MN	3+		4 PMN/MN	4++	
	4 NI	4−		0 MN	0+	
				2 NI	2−	

Histopathology in the lung samples from mice 5 days after infection with mucoid (0.8 × 10^6^ CFU per mouse) or non-mucoid (4 × 10^6^ CFU per mouse) *Pseudomonas aeruginosa*. Lung slides stained with HE were analyzed with respect to type of inflammation [acute type PMN (>90% PMN), chronic type (>90% MN), both types present, neither dominating PMN/MN or no inflammation observed (NI)] and degree of inflammation [+++ (severe inflammation and/or necrosis), ++ (moderate to severe focal inflammation), + (mild focal inflammation) or 0 (no inflammation observed)]. In addition, presence of atelectasis or micro-abscesses was noted. The number of mice with a certain type and degree of inflammation is presented. Likewise, the number of mice with atelectasis or micro-abscesses is presented. An inflammation involving PMNs were observed in significantly more mice infected with an early isolate (^*^p<0.05). In addition, a higher degree of inflammation was observed in mice infected with an early isolate, although the difference between 1988 and 2003 did not reach the level of significance (^**^p<0.02 for 1980 and ^***^p<0.0002 for 1988).

Mice infected with the late mucoid *P. aeruginosa* isolate from 2003 had significantly more often a PMN involving inflammation as well as significantly higher degree of inflammation (^****^p<0.02).

PMN, polymorphonuclear neutrophil granulocytes; MN, mononuclear cells; NI, no inflammation; CFU, colony-forming units; HE, haematoxylin and eosin.

#### Mucoid isolates

In contrast to mice infected with the non-mucoid isolates, the most severe histopathology was observed in mice infected with the late 2003 isolate. PMN-involved inflammation was observed in seven out of nine mice infected with the 2003 isolate as compared with none out of nine and two out of nine infected with the 1997 or the 1988 isolate, respectively (p<0.01 and p<0.05; Table 2). Three of the mice infected with the 2003 isolate even had a PMN-dominated inflammation. Moreover, an increased degree of inflammation was observed in the group of mice infected with the 2003 isolate as compared with the 1988 and 1997 groups (p<0.002 and p<0.02; Table 2), and micro-abscesses were only observed in mice infected with the 2003 isolate.

In a number of the mice infected with the 2003 mucoid isolate, the biofilm-embedded bacteria and the induced inflammation had spread to the periphery of the lungs, namely the alveoli lining the pleural membrane, in accordance with the observation that the chronic *P. aeruginosa* lung infection in CF patients is also a disease of the smaller airways (Fig. 3A) (29). In addition, PMNs invading the airway lumen could be seen in (Fig 3A). To support that it was actually *P. aeruginosa* that was seen in the peripheral airways of a fraction of mice infected with the late mucoid 2003 isolate, staining with PNA-FISH specific for *P. aeruginosa* was performed (Fig. 3G). The bacteria-like structures were specifically stained with the PNA-FISH, confirming the origin of the structures. No such staining was seen in areas without bacteria-like structures. Another striking feature was the observation of large, round inflammatory cells or foamy cells in some of the alveoli from mice infected with the 2003 isolate (Fig. 3B). In those areas, bacteria-like structures were not identified.

Comparisons between groups infected with the mucoid and the non-mucoid isolates were not carried out because the infection dose was reduced five times in mice infected with the mucoid isolates. However, it is notable that with this reduced infection dose, the histopathology in the group infected with the 2003 mucoid isolate was the worse of all groups.

### Cytokines

#### Non-mucoid isolates

**Fig. 4:**
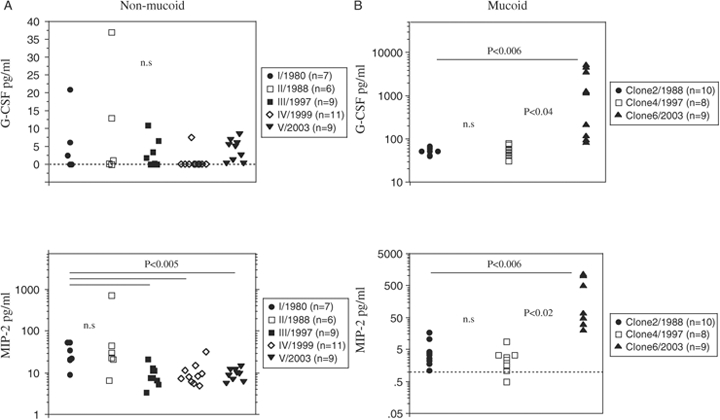
Pulmonary granulocyte colony-stimulating factor (G-CSF) and macrophage inflammatory protein-2 (MIP-2) production. Pulmonary production of G-CSF and MIP-2 in mice at day 5 after infection with non-mucoid [4 × 10^6^ colony-forming units (CFU) per mouse, panel A) or mucoid (0.8 × 10^6^ CFU per mouse, panel B) *Pseudomonas aeruginosa* isolates from different periods of chronic lung infection in a cystic fibrosis patient. Measured in supernatants from homogenized lungs. In mice infected with the non-mucoid isolates, the highest production of the inflammatory markers was observed in the groups infected with the early isolates (p<0.005 for MIP-2; NS for G-CSF). In contrast, only mice infected with late mucoid isolate showed a significant pulmonary G-CSF and MIP-2 response (p<0.02 for MIP-2 and G-CSF).

Although the highest levels of G-CSF were measured in mice infected with the early isolates, this did not reach statistical significance (Fig. 4A). The highest levels of MIP-2 were measured in the lung homogenates from mice infected with one of the early isolates from 1980 or 1988. The MIP-2 levels were significantly increased in the lungs of the 1980 group as compared with the 1997, 1999 and 2003 groups (p<0.005; Fig. 4A).

#### Mucoid isolates

The G-CSF levels were significantly increased in the lung homogenates from mice infected with the late 2003 isolate as compared with mice infected with the 1997 or the 1988 isolate (p<0.04 and p<0.02, respectively; Fig. 4B). Likewise, the levels of MIP-2 in the lung homogenates from mice infected with the 2003 isolate were approximately 10–100-fold higher than in mice infected with the 1997 or the 1988 isolate (p<0.02 and p<0.006, respectively; Fig. 4B). No significant differences in either G-CSF or MIP-2 levels in the lung homogenates between mice infected with the 1997 or the 1988 isolates were observed (Fig. 4B).

## DISCUSSION

The present serial animal experiments were undertaken with early, intermediate and late isolates of *P. aeruginosa*, with the same genotype based on identical PFGE patterns, causing chronic lung infection for decades in one CF patient. The reason for this approach was that chronic lung infections in mice cannot be maintained for more than 2–3 weeks. Using this concept, the study revealed differences in pathogenicity between the mucoid and the non-mucoid isolates. In addition, an evolution of virulence was observed. Non-mucoid isolates seemed to reduce their virulence, whereas mucoid isolates increased or at least maintained their virulence.

Rodents seem to be naturally resistant to *P. aeruginosa* lung infections and planktonic growing bacteria are easily cleared from the mice, except when mice are given a lethal infection dose. Therefore, in the majority of animal models of chronic *P. aeruginosa* lung infection embedded bacteria in either agar, agarose, seaweed or their own native alginate are used (27, 28, 30). However, even those models have a limited duration of 2–3 weeks (28, 30). In other models this problem is overcome by repeated exposure of bacteria; however, mice either succumb to or become resistant to this procedure (31, 32). Adding *P. aeruginosa* to the drinking water resulting in aspiration of bacteria to the lower airways in a fraction of the mice, or installing a plastic tube colonized with *P. aeruginosa* in a main bronchus, can significantly increase the duration of the infection in mice (33, 34). However, these alternatives are still limited to the life span of the mouse at the most extreme, and only investigate one single isolate, often being the sequenced laboratory strain PAO1. None of those models can reflect the 20–30 years of bacterial exposure to host responses and antibiotics, which, from a bacterial perspective, may represent as much as 65 000 generations (35). From a human perspective, this corresponds to 2.5 million years or the times of existence of *Homo erectus* that preceded *H. sapiens*.

Indeed, significant adaptations were observed in the present study. No significant difference in the virulence between the non-mucoid and the mucoid early isolates was observed. This corresponds with the observations that CF patients often are colonized recurrently with non-mucoid strains in the lungs before onset of the chronic lung infection (6). The shift to the mucoid phenotype is caused by mutations in the *muc* genes that function as AlgT repressors (36–38). Activated PMNs have been shown to be able to cause such mutations (39). Therefore, repeated infections with non-mucoid isolates inducing inflammation to a certain degree may be able to induce mutations resulting in mucoidy, and the two early non-mucoid isolates did induce significant inflammation. A control experiment with similar sets of phenotypic non-mucoid sequential *P. aeruginosa* from two other CF patients confirmed our findings of highest virulence in the early isolates (data not shown). In agreement, non-mucoid phenotypic sets of *P. aeruginosa* clones isolated during different time periods of the chronic lung infections in a number of CF patients were analysed for their ability to generate *in vitro* biofilms, hypermutability, colony morphology, motility, QS status and production of virulence factors (22). The early isolates harboured most of these abilities, and presumably would be the most virulent (25).

In later stages of the chronic lung infection, the differences in virulence between simultaneously isotypic mucoid and non-mucoid isolates became distinct. Whereas the virulence and the ability to establish a chronic lung infection of the non-mucoid isolates seemed to decline, the virulence of the two late mucoid isotypes increased and the infection dose had to be reduced five times. Using the reduced infection dose, the course of the infection became benign in mice infected with the intermediate 1997 isolate, whereas the majority of mice infected with the late 2003 isolate could not eliminate the infection, and severe inflammation was induced. Therefore, although the presented results indicate that only the mucoid 2003 isolate had the ability to generate infection and inflammation, the virulence was gradually increasing from the early mucoid 1988 isolate as demonstrated by infection with a higher infection dose. Furthermore, similar findings of increasing virulence were observed for two other clinical sets of mucoid sequential early and late *P. aeruginosa* isolates. The sequentially increasing differences in the virulence of mucoid and non-mucoid isolates were best evaluated by the histopathology, especially with the observation of the late mucoid strain generating inflammation and infection in the alveoli, whereas the non-mucoid strains almost exclusively infected more central airways. This phenomenon is interesting because the beads embedding mucoid or non-mucoid isolates are similar in size (median diameter 60 μm) (27) and therefore will be arrested in the larger airways during challenge (40). However, likewise, in the earlier studies from our group, only mucoid strains were capable of proceeding to the smaller airways of the lungs (28, 30). Furthermore, in the small, peripheral airways of the lungs, the bacteria generate stronger inflammatory responses because they are in contact with both residing alveolar macrophages and are in close contact with the capillaries, from where the inflammatory cells migrate into the airspace (41). Experimentally, this supports the theory that mucoid strains primarily reside in the peripheral, respiratory zones of the lungs, in contrast to the non-mucoid strains, which probably mainly reside in the larger, conductive airways (6).

The groups of mice with the more severe course of the infection, which were the groups infected with the early non-mucoid strains or the late mucoid strains, also showed the highest production of MIP-2 (a murine IL-8 analogue) and the important PMN mobilizer G-CSF. These observations correspond with the observation in CF patients where IL-8 is considered an important PMN chemoattractant, and G-CSF correlates with poor lung function and induction of a Th2-dominated response (42, 43). Whether these clones also induce a Th2-dominating response in mice remains to be investigated. Moreover, the maintained C12 production of the late mucoid isolate is in line with the observation that C12 can function as a chemoattractant, either directly or through induction of IL-8 (44).

A striking new observation during the histopathologic evaluation was the presence of swollen or foamy inflammatory cells located inside the alveoli in a fraction of the mice infected with the late mucoid 2003 isolate. The cause of the swollen cells is unknown, but they could be macrophages with a foamy appearance, indicating lipid phagocytosis due to necrotic PMNs. This phenomenon requires further investigation.

QS has previously been reported to be an important control system of virulence in *P. aeruginosa* lung infections, but also in extrapulmonary *P. aeruginosa* infections (45, 46). However, in contrast to the early isolates the late and most virulent mucoid strain from 2003 only produced the C12 homoserine lactone, suggesting a QS-independent virulence factor taking over infection control during the year-long adaptation of the chronic lung infection. Because alginate production is independent of QS, this may be the one decisive factor enabling *P. aeruginosa* to remain in the CF lungs at later stages of the disease (47). In a follow-up study by Lee and colleagues, it was indeed observed that the mucoid strains used in the present study maintained their ability to establish classic *in vitro* biofilms in the flowchamber system (B. Lee, personal communication). Moreover, previous animal studies have reported mucoidy of *P. aeruginosa* to be a pivotal virulence factor during lung infections (30, 48, 49).

Whether the distinct and changing virulence of non-mucoid and mucoid *P. aeruginosa* isotypes observed in the present study represents a general behaviour for chronic *P. aeruginosa* lung infections in CF has to be confirmed in future studies involving more isotypic sets of bacteria. However, involving two other sets of non-mucoid and mucoid isotypic *P. aeruginosa* confirmed our findings supporting the importance of involving the time perspective in animal models of chronic *P. aeruginosa* lung infections.

Diversification and adaptation has also been reported for diseases and conditions like *Helicobacter pylori* infections, characterized by year-long colonization of the gastric and duodenal mucosa resulting in ulcers, and with time, complications like cancer. During these periods of colonization, the strains undergo large genetic alterations through mutations and recombinations, resulting in host adaptations, but perhaps also altered virulence (50,51). Likewise, adaptations have been reported in *Porphyromonas gingivalis* colonizing the oral cavity for years, as well as *Streptococcus mutans* (52,53). Such intra-strain evolution can probably be demonstrated for other chronic colonizers in a stressful environment with the potential of becoming pathogenic. The present concept may also be applicable to model systems with those pathogens.

In conclusion, here we present a novel animal experimental strategy for investigation of year-long chronic infections and colonizations. Using this concept, important sequential differences in host responses were observed. Finally, our observations suggest a pivotal role for mucoidy in generating chronic *P. aeruginosa* lung infection in the peripheral airways of CF patients.

## References

[1] Matsui H, Grubb BR, Tarran R, Randell SH, Gatzy JT, Davis CW (1998). Evidence for periciliary liquid layer depletion, not abnormal ion composition, in the pathogenesis of cystic fibrosis airways disease. Cell.

[2] Hartl D, Griese M, Kappler M, Zissel G, Reinhardt D, Rebhan C (2006). Pulmonary T(H)2 response in Pseudomonas aeruginosa-infected patients with cystic fibrosis. J Allergy Clin Immunol.

[3] Høiby N, Flensborg EW, Beck B, Friis B, Jacobsen SV, Jacobsen L (1977). Pseudomonas aeruginosa infection in cystic fibrosis. Diagnostic and prognostic significance of Pseudomonas aeruginosa precipitins determined by means of crossed immunoelectrophoresis. Scand J Respir Dis.

[4] Moser C, Kjaergaard S, Pressler T, Kharazmi A, Koch C, Høiby N (2000). The immune response to chronic Pseudomonas aeruginosa lung infection in cystic fibrosis patients is predominantly of the Th2 type. APMIS.

[5] Costerton JW (2001). Cystic fibrosis pathogenesis and the role of biofilms in persistent infection. Trends Microbiol.

[6] Bjarnsholt T, Jensen PØ, Fiandaca MJ, Pedersen J, Hansen CR, Andersen CB *Pseudomonas aeruginosa* biofilms in the respiratory tract of cystic fibrosis patients. Pediatr Pulmonol.

[7] Ciofu O, Riis B, Pressler T, Poulsen HE, Høiby N (2005). Occurrence of hypermutable Pseudomonas aeruginosa in cystic fibrosis patients is associated with the oxidative stress caused by chronic lung inflammation. Antimicrob Agents Chemother.

[8] Oliver A, Canton R, Campo P, Baquero F, Blázquez J (2000). High frequency of hypermutable Pseudomonas aeruginosa in cystic fibrosis lung infection. Science.

[9] Döring G, Wörlitzsch D (2000). Inflammation in cystic fibrosis and its management. Paediatr Respir Rev.

[10] Rao S, Grigg J (2006). New insights into pulmonary inflammation in cystic fibrosis. Arch Dis Child.

[11] Jelsbak L, Johansen HK, Frost AL, Thøgersen R, Thomsen LF, Ciofu O (2007). Molecular epidemiology and dynamics of Pseudomonas aeruginosa populations in the lungs of cystic fibrosis patients. Infect Immun.

[12] Lozupone CA, Knight R (2007). Global patterns in bacterial diversity. Proc Natl Acad Sci USA.

[13] Nidelet T, Kaltz O (2007). Direct and correlated responses to selection in a host–parasite system: testing for the emergence of genotype specificity. Evolution Int J Org Evolution.

[14] Fukami T, Beaumont HJE, Zhang X-X, Rainey PB (2007). Immigration history controls diversification in experimental adaptive radiation. Nature.

[15] Harrison F, Buckling A (2007). High relatedness selects against hypermutability in bacterial metapopulations. Proc R Soc B.

[16] Rainey PB, Rainey K (2003). Evolution of cooperation and conflict in experimental bacterial populations. Nature.

[17] Spiers AJ, Buckling A, Rainey PB (2000). The causes of Pseudomonas diversity. Microbiology.

[18] Chugani S, Greenberg P (2006). The influence of human respiratory epithelia on Pseudomonas aeruginosa gene expression. Microbial Pathog.

[19] D'Argenio DA, Wu M, Hoffman LR, Kulasekara HD, Déziel E, Smith E (2007). Growth phenotypes of Pseudomonas aeruginosa lasR mutants adapted to the airways of cystic fibrosis patients. Mol Microbiol.

[20] Eberl L, Tümmler B (2004). Pseudomonas aeruginosa and Burkholderia cepacia in cystic fibrosis: genome evolution, interactions and adaptation. Int J Med Microbiol.

[21] Stover CK, Pham XQ, Erwin AL, Mizuguchi SD, Warrener P, Hickey MJ (2000). Complete genome sequence of Pseudomonas aeruginosa PAO1, an opportunistic pathogen. Nature.

[22] Lee B, Haagensen JAJ, Ciofu O, Andersen JB, Høiby N, Molin S (2005). Heterogeneity of biofilms formed by nonmucoid Pseudomonas aeruginosa isolates from patients with cystic fibrosis. J Clin Microbiol.

[23] Wörlitzsch D, Tarran R, Ulrich M, Schwab U, Cekici A, Meyer KC (2002). Effects of reduced mucus oxygen concentration in airway Pseudomonas infections of cystic fibrosis patients. J Clin Invest.

[24] Ojeniyi B, Petersen US, Høiby N (1993). Comparison of genome fingerprinting with conventional typing methods used on Pseudomonas aeruginosa isolates from cystic fibrosis patients. APMIS.

[25] Tenover FC, Arbeit RD, Goering RV, Mickelsen PA, Murray BE, Persing DH (1995). Interpreting chromosomal DNA restriction patterns produced by pulsed-field gel electrophoresis: criteria for bacterial strain typing. J Clin Microbiol.

[26] Hentzer M, Riedel K, Rasmussen TB, Heydorn A, Andersen JB, Parsek MR (2002). Inhibition of quorum sensing in Pseudomonas aeruginosa biofilm bacteria by a halogenated furanone compound. Microbiology.

[27] Pedersen SS, Shand GH, Hansen BL, Hansen GN (1990). Induction of experimental chronic Pseudomonas aeruginosa lung infection with P. aeruginosa entrapped in alginate microspheres. APMIS.

[28] Moser C, Johansen HK, Song Z, Hougen HP, Rygaard J, Høiby N (1997). Chronic Pseudomonas aeruginosa lung infection is more severe in Th2 responding BALB/c mice compared to Th1 responding C3H/HeN mice. APMIS.

[29] Farrel PM, Li Z, Kosorok MR, Laxova A, Green CG, Collins J (2003). Bronchopulmonary disease in children with cystic fibrosis after early or delayed diagnosis. Am J Respir Crit Care Med.

[30] Hoffmann N, Rasmussen TB, Jensen PØ, Stub C, Hentzer M, Molin S (2005). Novel mouse model of chronic Pseudomonas aeruginosa lung infection mimicking cystic fibrosis. Infect Immun.

[31] Moser C, Jensen PØ, Kobayashi O, Hougen HP, Song Z, Rygaard J (2002). Improved outcome of chronic Pseudomonas aeruginosa lung infection is associated with induction of a Th1-dominated cytokine response. Clin Exp Immunol.

[32] Van Heeckeren AM, Schluchter MD, Xue W, Davies PB (2006). Response to acute lung infection with mucoid Pseudomonas aeruginosa in cystic fibrosis. Am J Respir Crit Care Med.

[33] Coleman FT, Mueschenborn S, Meluleni G, Ray C, Carey VJ, Vargas SO (2003). Hypersusceptibility of cystic fibrosis mice to chronic Pseudomonas aeruginosa oropharyngeal colonization and lung infection. Proc Natl Acad Sci USA.

[34] Yanagihara K, Tomono K, Sawai T, Hirata Y, Kadota J, Koga T (1997). Effect of clarithromycin on lymphocytes in chronic respiratory Pseudomonas aeruginosa infection. Am J Respir Crit Care Med.

[35] Yang L, Haagensen JAJ, Jelsbak L, Johansen HK, Sternberg C, Høiby N (2008). In situ growth rates and biofilm development of Pseudomonas aeruginosa populations in chronic lung infections. J Bacteriol.

[36] Bragonzi A, Wörlitzsch D, Pier GB, Timpert P, Ulrich M, Hentzer M (2005). Nonmucoid Pseudomonas aeruginosa expresses alginate in the lungs of patients with cystic fibrosis and in a mouse model. J Infect Dis.

[37] Ciofu O, Fussing V, Bagge N, Koch C, Høiby N (2001). Characterization of paired mucoid/non-mucoid Pseudomonas aeruginosa isolates from Danish cystic fibrosis patients: antibiotic resistance, beta-lactamase activity and RiboPrinting. J Antimicrob Chemotherapy.

[38] Yoon SS, Coakley R, Lau GW, Lymar SV, Gaston B, Karabulut AC (2006). Anaerobic killing of mucoid Pseudomonas aeruginosa by acidified nitrite derivatives under cystic fibrosis airway conditions. J Clin Invest.

[39] Mathee K, Ciofu O, Sternberg C, Lindum PW, Campbell JI, Jensen P (1999). Mucoid conversion of Pseudomonas aeruginosa by hydrogen peroxide: a mechanism for virulence activation in the cystic fibrosis lung. Microbiology.

[40] West JB, Anthony R (2001). Coal workers' pneumoconiosis. Pulmonary Physiology and Pathophysiology. An Integrated, Case-Based Approach.

[41] Hagg JC (2004). Pathophysiology of airflow limitations in chronic obstructive pulmonary disease. Lancet.

[42] Jensen PØ, Moser C, Kharazmi A, Pressler T, Koch C, Høiby N (2006). Increased serum concentrations of G-CSF in cystic fibrosis patients with chronic Pseudomonas aeruginosa pneumonia. J Cyst Fibros.

[43] Moser C, Jensen PØ, Pressler T, Frederiksen B, Lanng S, Kharazmi A (2005). Serum concentrations of GM-CSF and G-CSF correlate with the Th1/Th2 cytokine response in cystic fibrosis patients with chronic Pseudomonas aeruginosa lung infection. APMIS.

[44] Zimmermann S, Wagner C, Müller W, Brenner-Weiss G, Hug F, Prior B (2006). Induction of neutrophil chemotaxis by the quorum-sensing molecule N-(3-oxododecanoyl)-l-homoserine lactone. Infect Immun.

[45] Bjarnsholt T, Jensen PØ, Rasmussen TB, Christophersen L, Calum H, Hentzer M (2005). Garlic blocks quorum sensing and promotes rapid clearing of pulmonary Pseudomonas aeruginosa infections. Microbiology.

[46] Christensen LD, Moser C, Jensen PØ, Rasmussen TB, Christophersen L, Kjelleberg S (2007). Impact of Pseudomonas aeruginosa quorum sensing on biofilm persistence in an in vivo intraperitoneal foreign-body infection model. Microbiology.

[47] Pedersen SS (1992). Lung infection with alginate-producing, mucoid Pseudomonas aeruginosa in cystic fibrosis. APMIS.

[48] Song Z, Wu H, Ciofu O, Kong KF, Høiby N, Rygaard J (2003). Pseudomonas aeruginosa alginate is refractory to Th1 immune response and impedes host immune clearance in a mouse model of acute lung infection. J Med Microbiol.

[49] Yu H, Hanes M, Chrisp CE, Boucher JC, Deretic V (1998). Microbial pathogenesis in cystic fibrosis: pulmonary clearance of mucoid Pseudomonas aeruginosa and inflammation in a mouse model of repeated respiratory challenge. Infect Immune.

[50] Salama NR, Gonzales-Valencia G, Deatherage B, Aviles-Jimenez F, Atherton JC, Graham DY (2007). Genetic analysis of Helicobacter pylori strain populations colonizing the stomach at different times postinfection. J Bacteriol.

[51] Suerbaum S, Josenhans C (2007). Helicobacter pylori evolution and phenotypic diversification in a changing host. Nat Rev Microbiol.

[52] Deng DM, ten Cate JM, Crielaard W (2007). The adaptive response of Streptococcus mutans towards oral care products: involvement of the ClpP serine protease. Eur J Oral Sci.

[53] Wang M, Shakhatreh M-AK, James D, Liang S, Nishiyama S, Yoshimura F (2007). Fimbrial proteins of Porphyromonas gingivalis mediate in vivo virulence and exploit TLR2 and complement receptor 3 to persist in macrophages. J Immunol.

